# Human CD34^+^ CD133^+^ Hematopoietic Stem Cells Cultured with Growth Factors Including Angptl5 Efficiently Engraft Adult NOD-SCID Il2rγ^−/−^ (NSG) Mice

**DOI:** 10.1371/journal.pone.0018382

**Published:** 2011-04-29

**Authors:** Adam C. Drake, Maroun Khoury, Ilya Leskov, Bettina P. Iliopoulou, Maria Fragoso, Harvey Lodish, Jianzhu Chen

**Affiliations:** 1 Koch Institute for Integrative Cancer Research, Department of Biology, Massachusetts Institute of Technology, Cambridge, Massachusetts, United States of America; 2 Interdisciplinary Research Group in Infectious Diseases, Singapore-MIT Alliance for Research and Technology (SMART), Singapore, Singapore; 3 Whitehead Institute for Biomedical Research, Cambridge, Massachusetts, United States of America; Beckman Research Institute of the City of Hope, United States of America

## Abstract

Increasing demand for human hematopoietic stem cells (HSCs) in clinical and research applications necessitates expansion of HSCs *in vitro*. Before these cells can be used they must be carefully evaluated to assess their stem cell activity. Here, we expanded cord blood CD34^+^ CD133^+^ cells in a defined medium containing angiopoietin like 5 and insulin-like growth factor binding protein 2 and evaluated the cells for stem cell activity in NOD-*SCID Il2rg^−/−^* (NSG) mice by multi-lineage engraftment, long term reconstitution, limiting dilution and serial reconstitution. The phenotype of expanded cells was characterized by flow cytometry during the course of expansion and following engraftment in mice. We show that the SCID repopulating activity resides in the CD34^+^ CD133^+^ fraction of expanded cells and that CD34^+^ CD133^+^ cell number correlates with SCID repopulating activity before and after culture. The expanded cells mediate long-term hematopoiesis and serial reconstitution in NSG mice. Furthermore, they efficiently reconstitute not only neonate but also adult NSG recipients, generating human blood cell populations similar to those reported in mice reconstituted with uncultured human HSCs. These findings suggest an expansion of long term HSCs in our culture and show that expression of CD34 and CD133 serves as a marker for HSC activity in human cord blood cell cultures. The ability to expand human HSCs *in vitro* should facilitate clinical use of HSCs and large-scale construction of humanized mice from the same donor for research applications.

## Introduction

Human hematopoietic stem cells (HSCs) were the first clinically important class of stem cells and are now frequently used in autologous and heterologous transplantation to treat a range of hematologic malignancies and congenital immunological defects [1], [2]. HSCs for transplantation are usually isolated from umbilical cord blood, bone marrow (BM), or mobilized adult peripheral blood [3], [4]. In the case of cord blood, where sample sizes are small, adults often receive cells from two donors to ensure infusion of sufficient numbers of HSCs. The chronic shortage of well matched HSCs has stimulated significant interest in expanding HSCs *in vitro* for transplantation.

There are three major challenges in expanding human HSCs for clinical and preclinical applications. First, efficient methods are needed for expanding HSCs rather than allowing cells to proliferate, differentiate and eventually die. Over the last two decades, quite a few HSC expansion protocols have been reported. The earlier studies explored the use of stromal cells as ‘feeders’ [5]. More recently feeder-free cultures using defined media and recombinant growth factors were used because of their simplicity and increasing evidence of efficacy [6]. Second, the expanded cells have to be characterized and their stem cell activity quantified. The best evaluations require engraftment into immunodeficient mice, such as NOD-scid mice, and assaying for long-term multi-lineage reconstitution and serial reconstitution [7]. Recently, better mouse strains, such as NOD-*scid Il2rg^−/−^* (NSG) mice, that lack their own T cells, B cells and natural killer (NK) cells [8], [9], [10], [11] have been used because these mice support both long-term and much more robust lymphoid and myeloid cell reconstitution, resulting in mice with robust enough grafts to begin studying the human cells (humanized mice). Third, there is a need for surrogate markers for rapid assessment of HSC expansion. This is because the functional verification by engrafting HSCs in immunodeficient mice takes months. The availability of surrogate markers that can quickly assess the quality of expanded cells make it possible to use the expanded cells immediately without freezing the cells and waiting for the *in vivo* results. By a combination of fractionation and *in vivo* reconstitution, studies have identified cell surface markers that differentiate freshly isolated HSCs from more committed progenitors in both humans and mice [7]. However, identification of HSCs following expansion is complicated by changes in both surface marker expression and stem cell activity during the culture. To our knowledge, surface markers that closely correlate with SCID repopulating activity both before and after HSC culture have not been reported.

We have previously shown that stem cell factor (SCF), thrombopoietin (TPO) and fibroblast growth factor 1 (FGF1) in combination with angiopoietin-like proteins or insulin-like growth factor binding protein 2 (IGFBP2) support a significant expansion of murine HSCs in a feeder-cell-free, serum-free culture [12], [13]. Further study demonstrated that the combination of these five growth factors also supports expansion of human cord blood CD34^+^ CD133^+^ cells. When the expanded human cells were tested for the ability to reconstitute NOD-scid recipient mice, an approximately 20-fold expansion of SCID repopulating cells (SRCs) was obtained during a ten-day culture [14].

In this report, we expand cord blood CD34^+^ CD133^+^ cells in the defined medium described above and assess the cell surface phenotype during the course of the culture and the stem cell activity of resulting cells in NSG recipient mice. Specifically, we address three questions. Firstly, what cell surface phenotype correlates with HSC activity before and after *in vitro* expansion? Secondly, does the expansion of SRCs observed in NOD-SCID mice occur in NSG mice and if so does this expansion correlate with an expansion of HSCs? Lastly, can the expanded cells be used for large-scale construction of humanized mice for preclinical studies?

## Materials and Methods

### Ethics Statement

All research with human samples and mice was performed in compliance with the institutional guidelines, the World Medical Association's Decleration of Helsinki and the US Department of Health and Human Services Guide for the Care and use of Laboratory Animals. The MIT committee on the use of humans as experimental subjects (MIT COUHES) granted the research a waiver as all human samples (umbilical cord blood, normally disposed of as medical waste) were collected anonymously with parental consent by a 3rd party and purchased from that party for the research. The MIT committee on animal care (MIT CAC) approved the research as part of animal protocol 0310-027-13.

### Cell isolation and culture

Purified CD133^+^ human umbilical cord HSCs were purchased from AllCells (California) or National Disease Research Interchange (NDRI). Alternatively, CD133^+^ cells were purified from fresh umbilical cord blood purchased from NDRI. Umbilical cord blood was centrifuged through ficoll-hypaque, and purified using a combination of PE-conjugated anti-CD133 antibody (Miltenyi) and PE positive selection kit (Stem Cell Technologies) according to the manufacturers' protocols. Human HSCs were cultured as reported previously [14], using StemSpan (Stem Cell Technologies, Vancouver) supplemented with human growth factors: 10 ng/ml FGF-1 (Invitrogen), 20 ng/ml SCF, 10 ng/ml TPO, 100 ng/ml IGFBP2 (all R&D systems), 500 ng/ml Angiopoietin like 5 (Abnova, Taiwan), 10 micrograms/ml of heparin and 1× penicillin/streptomycin (Mediatech, Manassas, VA). Briefly, CD133^+^ cells were plated at 50,000 cells/ml in growth factor-supplemented StemSpan in a 96 well round bottomed plate, at 200 µl/well. Cells were transferred to a 6 well plate 2–3 days later and more media was added to keep cell density below 200,000/ml one day after the transfer. Cells were supplemented with additional media 2–3 days later in order to dilute them to 700,000 cells/ml. Cells were then allowed to expand until the end of the 10 day culture.

### Mice

NOD-*SCID Il2rg^−/−^* mice were obtained from the Jackson Laboratory and bred in the animal facility at Massachusetts Institute of Technology. Neonate and adult mice were sub-lethally irradiated with 100 or 270 rads, respectively, using a Gamma Cell 40 Caesium source, as reported previously [15]. Expanded, re-isolated or unexpanded human HSCs were transferred into neonates by intracardiac injection within the first 48 hours after birth or into adult (2–4 month old) mice by tail vein injection.

### Tissue preparation, antibodies and flow cytometry

At various time points following injection of NSG recipients with human HSCs, mice were euthanized by CO_2_ asphyxiation and blood, bone marrow, spleen, lymph nodes and thymus were harvested. The spleens were digested by collagenase D at 37 degrees for 1 hour and the bone marrow were flushed using syringes with a 27 gauge needle. All organs were then disrupted by grinding between frosted glass cover slips and single cell suspensions were prepared. Samples were lysed of red blood cells and cells were counted. FITC, PE, PerCP/cy5.5, APC, PE/Cy7 or APC/Cy7 conjugated antibodies, including human CD3, CD4, CD8, CD10, CD11c, CD14, CD15, CD16, CD19, CD20, CD33, CD34, CD41, CD45, CD56, CD69, CD71, CD209, CD235ab, HLA-DR, and murine CD45.1, were from Biolegend. Anti-CD133 antibody was from Miltenyi. Cells were stained with appropriate combination of antibodies and then analyzed on FACScalibur, FACS-Canto or LSR II flow cytometers (Beckton Dickinson) in the MIT Koch Institute flow cytometry core facility. Dead cells were excluded from analysis by DAPI or propidium iodide staining.

### Limiting dilution assay

In order to calculate how many cells within a sample are needed to minimally repopulate a mouse (a repopulating unit) small numbers of cells (5000, 1000, or 400) were injected into sub-lethally irradiated NSG mice. Three to 4 months after injection, bone marrow was harvested and analyzed for the level of human cell reconstitution. A repopulating unit is calculated as the number of cells at which 37% of injections fail to give a minimal human cell engraftment in bone marrow. Based on Fishers exact test the thresholds selected and number of events collected (>100,000/mouse) give chance of false positives as less than 1e-9/mouse.

### Chimerism

Chimerism, or the level of human leukocyte reconstitution, was calculated as follows:

Chimerism = %CD45^+^ human cell/(%CD45^+^ human cell+%CD45^+^ mouse cell).

### Statistical analysis

Statistical analysis was carried out using Graphpad Prism 5. Two way ANOVA was used to compare series and the two tailed T test with Welch's correction were used for pairwise comparisons.

## Results

### Expansion of CD34^+^ CD133^+^ cells correlates with SCID-repopulating activity

**Figure 1:**
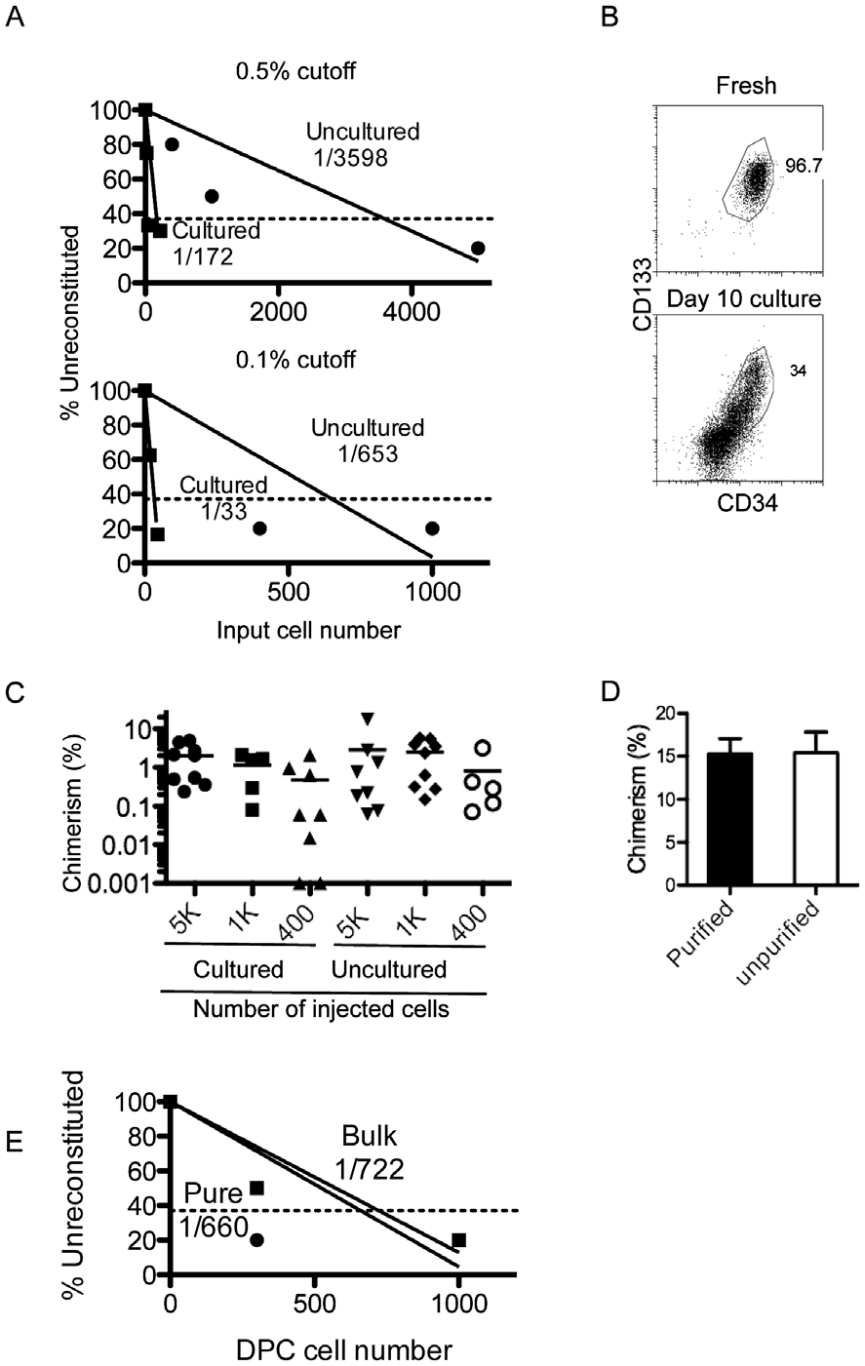
Expanded CD34^+^ CD133^+^ cells are SCID repopulating cells. (A) Limiting dilution analysis comparing cultured (squares) vs. uncultured (circles) cells for SRC frequency. CD133^+^ cord blood cells (5,000, 1,000 and 400) and their cultured progeny were injected into sub-lethally irradiated neonate recipients. Recipients were analyzed for human cell reconstitution in the bone marrow by staining for human CD45 and mouse CD45 followed by flow cytometry. SRC frequencies calculated by Poisson statistics are shown for each population based on number of input CD34^+^ CD133^+^ cells. Values for a 0.5% cutoff (top) and 0.1% cutoff (bottom) are shown. (B) Representative CD34 versus CD133 staining profiles of purified human HSCs at the start of in vitro expansion and cells after culture for 10 days. The number indicates percentage of CD34^+^ CD133^+^ cells in the gated region. (C) Comparison of bone marrow chimerism (human CD45 vs. mouse CD45.1) in sub-lethally irradiated neonate NSG recipients injected with 5000, 1000 and 400 cultured or uncultured CD34^+^ CD133^+^ cells. Horizontal bars show the mean, each symbol is an individual mouse. Two tail ANOVA testing shows no significant differences between data sets (p = 0.23). (D) Comparison of reconstitution in neonate NSG recipients transferred with 100,000 purified, cultured CD34^+^ CD133^+^ cells or total cultured cells (unpurified) containing 100,000 CD34^+^ CD133^+^ cells. Peripheral blood mononuclear cells were analyzed for human and mouse CD45 12 weeks after transfer. Mean ± SD is shown with 7 mice per group. (E) Limiting dilution analysis comparing purified (circles) and unpurified (squares) CD34^+^ CD133^+^ cells for SRC frequency. 300 and 1000 purified CD34^+^ CD133^+^ cells or total cells containing 300 and 1000 CD34^+^ CD133^+^ cells were transferred into sub-lethally irradiated adult NSG mice. Recipients were analyzed for human cell reconstitution in the bone marrow 8 weeks later by flow cytometry. Frequencies calculated using Poisson statistics are shown for each population based on the number of CD34^+^ CD133^+^ cells. All limiting dilutions represent 6–10 mice per data point and are single cultures representative of at least 2 independent experiments.

In order to assess how well the cultured progeny of CD133^+^ cells reconstitute NSG recipient mice small numbers of CD133^+^ cord blood cells (5,000, 1,000 and 400) and their cultured progeny were injected into sub-lethally irradiated neonate recipients. Human CD133^+^ cells were purified from cord blood and either injected into NSG mice or cultured for 10 days with SCF, TPO, FGF, IGFBP2 and angiopoietin-like 5 as previously described [14] and then injected into NSG mice. Three months later, mice were analyzed for human cell reconstitution in the bone marrow by staining for human CD45 and mouse CD45.1 followed by flow cytometry. In order to avoid any potential bias in the analysis, we employed two cutoffs of 0.1% and 0.5% of human cell reconstitution in the bone marrow that have been commonly used in recent publications [14], [16]. We determined the frequency of SRCs among total input cells using Poisson statistics for both cultured and uncultured cells at both thresholds (Figure 1A). At the 0.1% cutoff 653 input cells were needed for minimal repopulation and just 33 cells after 10 days of culture, an ∼20 fold increase in SCID repopulating activity following culture. At the 0.5% threshold 3598 uncultured cells were needed to minimally repopulate and the progeny of just 172 cells after 10 days of culture, an ∼21 fold increase in SCID repopulating activity. These results are similar to the ∼20 fold expansion of SCID repopulating activity that we reported for the expanded cells in NOD-*SCID* recipient mice.

Next we assessed the cell surface phenotype before and after culture. Starting populations of cells were 80–99% CD133^+^ with all of these cells also expressing CD34 (Figure 1B). Following 10 days of culture 13–34% of the expanded cells expressed CD34 and CD133 concurrently (Figure 1B). We reasoned that CD34^+^ CD133^+^ double positive cells might also identify the HSC-containing population after culture. Upon assessing the total number of these cells at the end of 10 day culture, we found that CD34^+^ CD133^+^ number increased 14–30 fold (mean 21 fold) although the increase in total cell number varied from 66–160 fold (Mean 110 fold). Using a single marker to identify the stem/progenitor population resulted in much more variable counts as CD133^+^ cell number expanded 10–70 fold (mean 28 fold) and CD34^+^ cells expanded 8–80 fold (mean 22 fold). In fact, almost all cells in the culture were CD34^dim^ or CD34^+^ on day 10 (data not shown), making it difficult to quantify them. In all cases CD34^+^ CD133^+^ cell number in the culture correlated tightly with SCID repopulating activity in mice. We therefore used the double marker CD34^+^ CD133^+^ as it gave a robust identification of the SCID repopulating cells whereas the single markers varied more from culture to culture due to poor separation in some cultures.

To assess the capacity of CD34^+^ CD133^+^ cell number to predict SCID repopulating activity before and after culture, 400, 1000 and 5000 uncultured CD34^+^ CD133^+^ cells and the total cultured cells containing 400, 1000 and 5000 CD34^+^ CD133^+^ cells were injected into neonate NSG mice and the chimerism in the bone marrow was assessed 12 weeks after injection. Similar levels of human cell reconstitution were observed at the three corresponding CD34^+^ CD133^+^ cell levels (Figure 1C). Furthermore, we purified CD34^+^ CD133^+^ cells from day 10 cultures by magnetic enrichment and injected these cells (1×10^5^ per recipient) or total cultured cells containing the same number of CD34^+^ CD133^+^ cells into NSG mice. Twelve weeks after transfer chimerism was assessed in the peripheral blood. Both groups of mice showed similar levels of long term reconstitution (Figure 1D). In contrast, when 30 million cultured cells depleted of CD34^+^ CD133^+^ cells were injected into sub-lethally irradiated adult mice, no reconstitution was detected in the peripheral blood (data not shown). To eliminate the possibility that non-CD34^+^ CD133^+^ cells while not able to directly engraft NSG mice might promote or inhibit engraftment of CD34^+^ CD133^+^ cells, following expansion we transferred 300 and 1000 purified CD34^+^ CD133^+^ cells or total cells containing 300 and 1000 CD34^+^ CD133^+^ cells into sub-lethally irradiated adult NSG mice. The mice were sacrificed 8 weeks later and assessed for minimal reconstitution in the bone marrow. As shown in Figure 1E, both purified and unpurified CD34^+^ CD133^+^ cells had similar repopulating activity regardless of the presence of other cells from the culture. These results show that the SCID repopulating activity resides in the CD34^+^ CD133^+^ population and is comparable before and after culture.

### Expanded CD34^+^ CD133^+^ cells maintain capacity for long term engraftment and serial reconstitution in NSG mice

**Figure 2:**
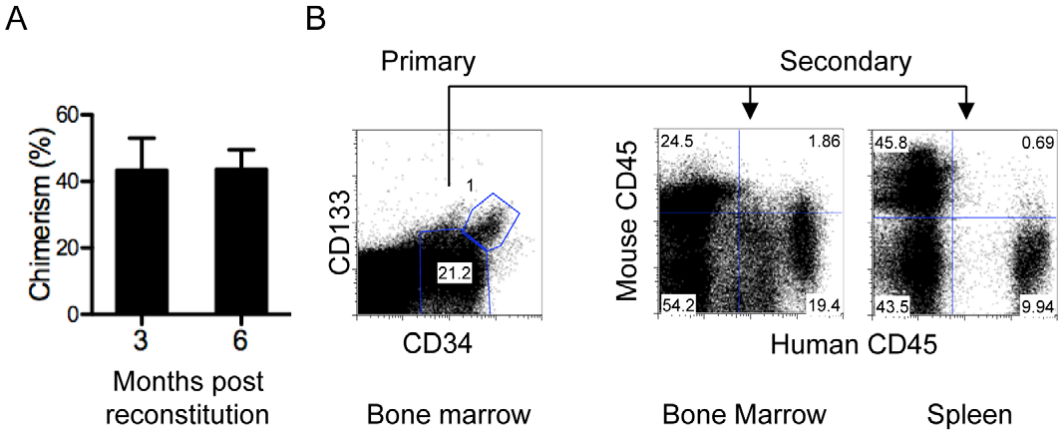
Expanded CD34^+^ CD133^+^ cells contain long-term HSCs. (A) Comparison of chimerism in peripheral blood at 3–4 and 6–7 months post reconstitution. Sub lethally irradiated adult NSG mice were injected with CD34^+^ CD133^+^ cells (2.5–5×10^5^ per mouse) 3–4 and 6–7 months post injection PBMCs were stained for human and mouse CD45. Mean ± SD is shown with 6 mice per group. (B) Presence of human CD34^+^ CD133^+^ cells in the bone marrow of reconstituted mice. Cells from the bone marrow of primary recipient mice 3–4 month after reconstitution were analyzed for human CD45, CD34 and CD133. Representative CD34 versus CD133 staining profiles are shown for human CD45^+^ cells. The number indicates the percentage of cells in the gated region. Serial reconstitution. Single cell suspension was prepared from the bone marrow of the primary recipient mice 14–28 weeks after engraftment. Human cells were enriched by depleting cells expressing mouse CD45 or by positive selection for human CD34^+^ cells by magnetic sorting. An equivalent of one femur's worth of enriched human cells was transferred into one sub-lethally irradiated secondary neonate or adult recipient. Twelve weeks later, cells from the spleen and bone marrow of secondary recipient mice were analyzed for human and mouse CD45. Human CD45 versus mouse CD45 staining profiles are shown for the secondary mouse with the highest human chimerism (n = 10, see Table 1).

**Figure 3:**
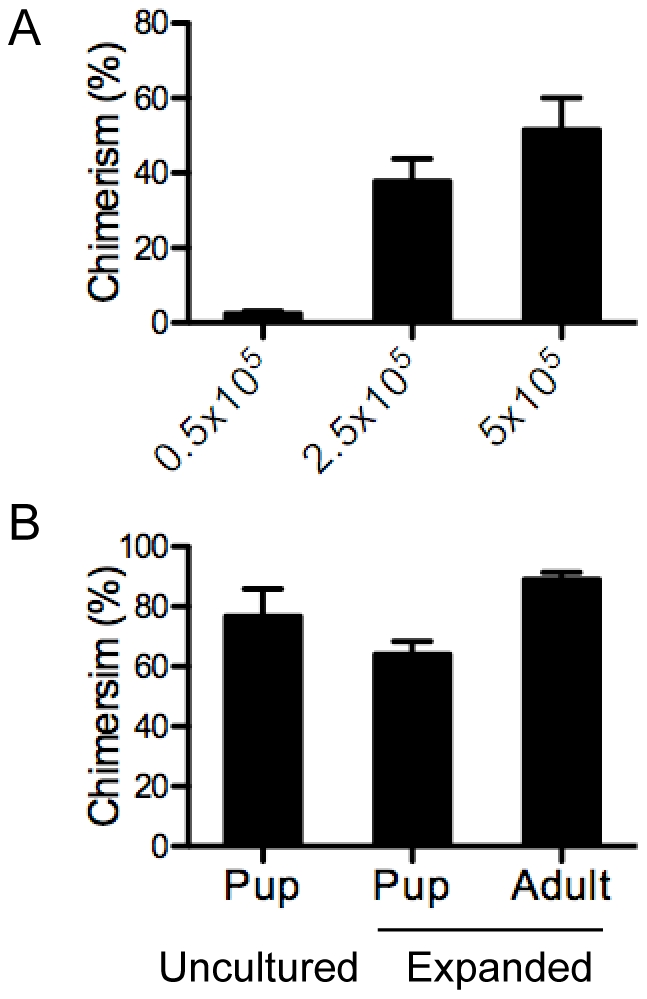
Expanded CD34^+^ CD133^+^ cells give robust reconstitution in both neonate and adult recipients. (A) Comparison of reconstitution levels in mice injected with different numbers of expanded cells. Sub-lethally irradiated adult NSG recipients were injected with cultured cells containing 0.5×10^5^, 2.5×10^5^ or 5×10^5^ CD34^+^ CD133^+^ cells. Eight weeks later, PBMCs were analyzed for human and mouse CD45 expression. Mean ± SD is shown, n = 6–8 per group. (B) Comparison of reconstitution levels in the bone marrow of neonate and adult recipients. Sub-lethally irradiated adult NSG recipients were injected with expanded cells containing 2.5×10^5^ CD34^+^ CD133^+^ cells. Sub-lethally irradiated neonates were injected with 1×10^5^ unexpanded or expanded CD34^+^ CD133^+^ cells. Twelve to sixteen week later, bone marrow was analyzed for human and mouse CD45 expression. Mean ± SD is shown, n = 4–6 per group.

**Figure 4:**
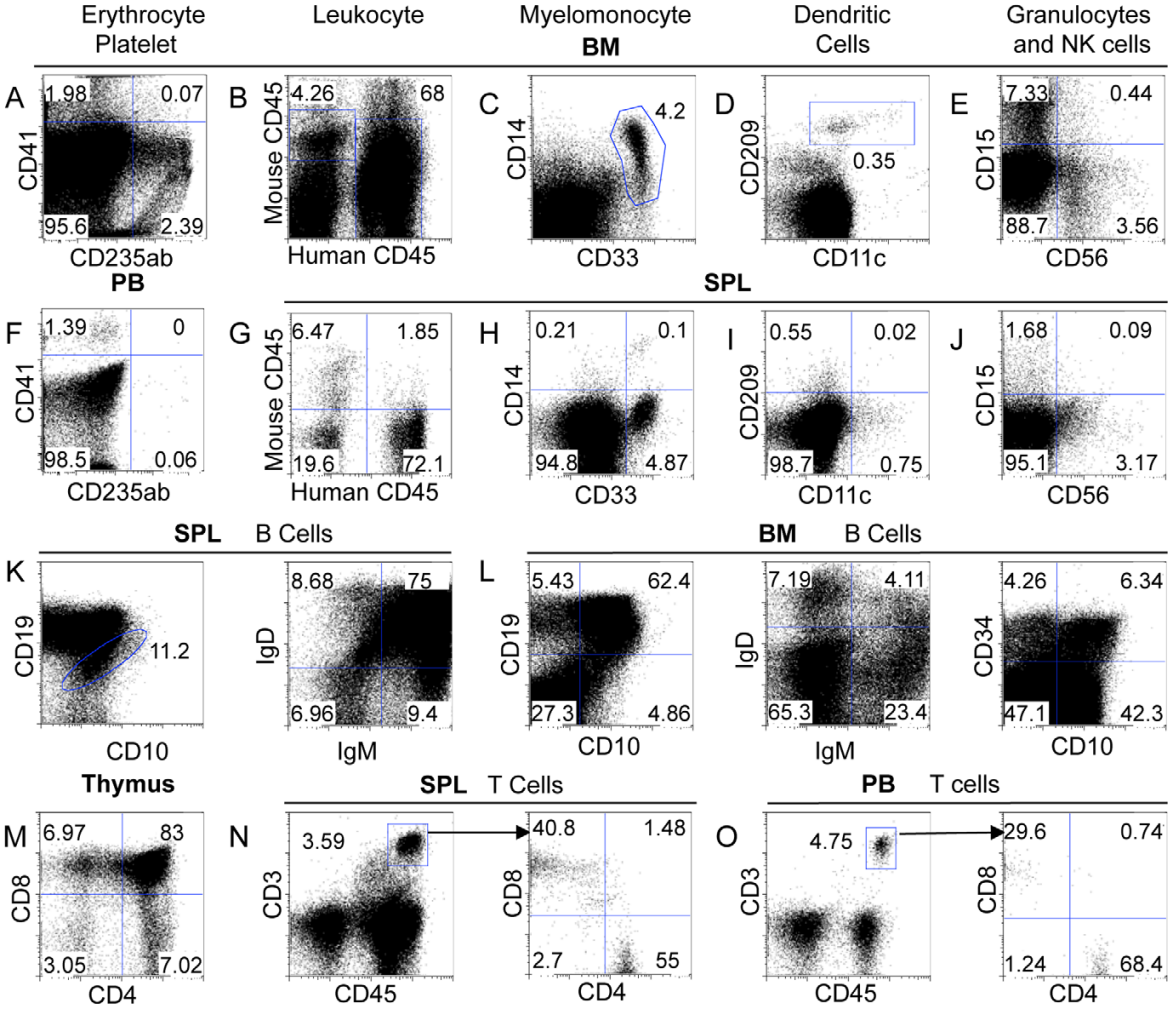
Multi-lineage reconstitution in adult NSG mice. Sub-lethally irradiated NSG adults were injected with 2.5–5×10^5^ expanded CD34^+^ CD133^+^ cells. Cells in the bone marrow (A–E, L), blood (F, O), thymus (M) and spleen (G–J, K, N) were analyzed by flow cytometry 12 weeks after reconstitution. Shows are staining profiles for erythrocytes (CD235ab^+^) and platelets (CD41^+^) (A and F), total leukocytes (human CD45^+^) (B and G), myelomonocytes (CD14^+^ CD33^+^) (C and H), dendritic cells (CD11c^+^ CD209^+^) (D and I), granulocytes (CD15^+^) and natural killer cells (CD56^+^) (E and J), B cell stages (proB - CD34^+^ CD10^+^, immature - CD10^+^, mature - IgM^+^ IgD^+^) (K and L), T cell precursors (double negative, double positive and single positive) (M) and mature T cells (CD45^+^ CD3^+^ expressing either CD4 or CD8) (N and O). The numbers indicate percentages of cells in the gated region. Representative data from one of at least 5 mice are shown. All cells shown are gated on live human cells (FSC/SSC/live/CD45^+^) except A, F (FSC/SSC only) B, G (FSC/SSC/live) and the IgM vs IgG stains (FSC/SSC/live/CD45^+^/CD19^+^). Summaries of human cell characterization can also be found in Table 2.

Human HSCs are characterized by the ability to support long-term (6 month or longer) as well as serial reconstitution in immune-deficient mice. As sublethally irradiated NSG mice do not develop lethal thymomas as NOD-scid mice do, we determined whether the SCID repopulating cells from the 10 day culture contain long-term HSCs. Thus, 2.5–5×10^5^ cultured CD34^+^ CD133^+^ cells were transferred into sub-lethally irradiated adult NSG mice. Three and six month later, peripheral blood mononuclear cells (PBMC) of recipient mice were analyzed for the presence of human CD45^+^ cells. As shown in Figure 2A the chimerism of these mice was statistically indistinguishable at 3 and 6 months post reconstitution. Furthermore, the number and lineage of human cells in the bone marrow, spleen, and thymus were similar between mice that were reconstituted for 3–4 months and for 6–7 months (Figures 3, 4 and data not shown). In addition, primary reconstituted NSG mice were sacrificed 3–6 months after the initial transfer and human cells were enriched either by depleting cells expressing mouse CD45 or by positive selection for human CD34^+^ cells. Each secondary recipient mouse was reconstituted with a fraction of enriched cells equivalent to those from 1 femur. Three months later, the secondary recipients were assessed for human cell chimerism in the bone marrow and spleen. When transferred human cells were depleted of mouse CD45^+^ cells, high levels (1.7–11.5%) of secondary reconstitution were observed (Figure 2 and Table 1), possibly reflecting the presence of human precursor cells in the transferred population rather than just stem cells. When transferred human cells were CD34-selected, significant levels of reconstitution (0.12–0.88%) were observed in all secondary recipients in three separate experiments (Table 1). Considering that 0.1% chimerism in the bone marrow is generally considered as positive secondary reconstitution with human HSCs, these results suggest that expanded CD34^+^ CD133^+^ cells contain long-term HSCs.

**Table 1:** Summary of secondary reconstitutions in NSG mice.

Exp #	HSC Origin	Primary Recipient	Length of Primary Reconstitution (wks)	Secondary Recipients	Method of HSC Enrichment	Chimerism (%)	#CD34+ injected
1	Cord #1	Adult	14	Adult	CD45.1 depleted	19.4	200,000
1	Cord #1	Adult	14	Adult	CD45.1 depleted	0.4	200,000
2	Cord #1	Adult	28	pup	CD45.1 depleted	2.05	80,000
2	Cord #1	Adult	28	pup	CD45.1 depleted	1.45	80,000
3	Cord #2	pup	14	Adult	CD34 Enrich	0.24	90,000
3	Cord #2	pup	14	Adult	CD34 Enrich	0.23	90,000
4	Cord #3	Adult	16	Adult	CD34 Enrich	0.59	50,000
4	Cord #3	Adult	16	Adult	CD34 Enrich	0.88	50,000
4	Cord #3	Adult	16	Adult	CD34 Enrich	0.13	50,000
5	Cord #4	Adult	16	Adult	CD34 Enrich	0.12	n.d.

2.5–5×10^5^ expanded CD34^+^ CD133^+^ cells were transferred into the primary neonate or adult NSG recipients. 14–28 weeks after transfer, single cell suspension was prepared from the bone marrow of recipient mice. Human cells were isolated by either depleting mouse CD45^+^ cells or enriching for human CD34^+^ cells and then transferred into secondary neonate or adult recipients. Each secondary recipient received an equivalent of one femur's worth of enriched human cells. The numbers of CD34^+^ cells were calculated and shown to the nearest 10,000 cells. n.d. – not determined. 12 weeks after secondary transfer, chimerism was assessed in the bone marrow by staining for human and mouse CD45.

### Expanded CD34^+^ CD133^+^ cells give robust reconstitution in both neonate and adult recipients

The significant expansion of CD34^+^ CD133^+^ cells makes it possible to reconstitute large numbers of adult mice from a single cord. To assess the capacity of expanded CD34^+^ CD133^+^ cells for reconstitution of adult recipients, increasing numbers of expanded cells were injected into sub-lethally irradiated adult NSG mice. The percentage of human cells (chimerism) in the peripheral blood was lower when 0.5×10^5^ CD34^+^ CD133^+^ cells were injected (Figure 3A). However, chimerism reached 40–60% when 2.5×10^5^ to 5×10^5^ CD34^+^ CD133^+^ cells were injected. For these reasons, 2.5–5×10^5^ CD34^+^ CD133^+^ cells were injected into adult recipients in the following studies.

We also compared reconstitution in the bone marrow and spleen of adult and neonate recipients injected with expanded or unexpanded cells. The degree of chimerism was similar in the bone marrow (62–78%) of neonate recipients injected with expanded or unexpanded CD34^+^ CD133^+^ cells (Figure 3B), consistent with a previous report [8]. The chimerism in the spleen of neonate recipients injected with expanded or unexpanded CD34^+^ CD133^+^ cells were also similar (∼80%, data not shown), though these values were slightly higher than those reported by Ishikawa et al. (∼55%) [8]. Surprisingly, the chimerism was ∼90% in both the bone marrow and spleen of adult recipients when 2.5–5×10^5^ CD34^+^ CD133^+^ cells were injected (Figure 3B and Table 2). Together, these results suggest that expanded CD34^+^ CD133^+^ cells are capable of robust reconstitution in both neonate and adult recipients.

**Table 2:** Summary of reconstitution of adult NSG mice injected with 2.5–5×10^5^ expanded CD34^+^CD133^+^cells.

Cell Type	Markers	Blood		Spleen		Bone Marrow	
		%	SD	%	SD	%	SD
Human Leukocyte	CD45+	56.7	21.7	92.3	2.3	89.0	5.3
HSCs	CD34^+^CD133^+^	N.D.	-	N.D.	-	1.0	0.3
Erythrocyte	CD235ab^+^	0.05	0.01	N.D.	-	1.1	0.9
Platelet	CD41^+^	0.4	0.7	N.D.	-	1.2	1.1
Myelomonocyte	CD14^+^CD33^+^	4.4	1.1	1.3	0.0	5.3	1.3
Dendritic Cell	CD11c^+^CD209^+^	N.D.	-	0.1	0.05	0.3	0.1
Gruanulocyte	CD15^+^	3.9	1.0	1.6	0.0	7.0	3.6
NK cell	CD56^+^	1.6	0.4	3.5	1.0	2.6	2.7
Pro B Cell	CD34^+^CD19^+^CD20^−^	N.D.	-	1.0	0.5	12.5	1.6
Pre B Cell	CD34^−^CD19^+^CD20^+^IgM^−^	N.D.	-	9.9	4.6	49.5	2.9
Immature B Cell	CD19^+^IgM^+^CD10^+^	N.D.	-	60.5	1.7	18.8	2.9
Mature B Cell	CD19^+^IgM^+^CD10^−^	N.D.	-	15.6	3.7	3.7	1.1
CD4 T Cell	CD3^+^CD4^+^	3.1	0.9	3.4	1.2	0.6	0.7
CD8 T Cell	CD3^+^CD8^+^	1.6	0.5	2.7	1.0	0.3	0.5
Circulating B Cell	IgM^+^CD19^+^	76.6	3.356	N.D.	-	N.D.	-

Data are a compilation of 24 mice. Each stain was performed on a minimum of 4 mice and all stains were performed on mice derived from at least two unrelated cords. All percentage calculations are based on the following gated groups: human leukocytes on total live (DAPI/PI negative) human or mouse CD45^+^ cells, erythrocytes and platelets on FSC versus SSC gate, and all other groups on live human CD45^+^ cells. Shown are mean percentage and SD of specific cell types in blood, spleen and bone marrow. ND, not determined.

### Human cell reconstitution in adult NSG mice reconstituted with expanded CD34^+^ CD133^+^ cells

We further characterized in detail the development of human blood lineage cells in adult NSG recipients injected with expanded CD34^+^ CD133^+^ cells. We assessed whether CD34^+^ CD133^+^ cells were present in the reconstituted mice. On average, 1% of the human cells in the bone marrow were CD34^+^ and CD133^+^ (Figure 2B and Table 2). We assessed the presence of various myeloid lineage cells, erythrocytes and platelets in the bone marrow and peripheral blood. The level of CD235ab^+^ human erythrocytes was low in the peripheral blood (∼0.05%) and slightly higher in the bone marrow (∼2%) (Figure 4A and F, Table 2), consistent with a previous report [8]. The level of CD41^+^ human platelets was also low in peripheral blood (∼0.4%) and higher in the bone marrow (∼1.2%) (Figure 4A and F, Table 2). A significant population of CD33^+^ CD14^+^ human myelomonocytes was detected in both bone marrow and spleen (Figure 4 C and H, Table 2), although the level of CD14 expression was significantly lower on those in the spleen than on those in the bone marrow. The pro-inflammatory subset of monocytes is known to express lower levels of CD14 and is CD38 negative [17]. A low level of CD209^+^ CD11c^+^ human dendritic cells was seen in the bone marrow, though these cells were almost completely absent from the spleen (Figure 4D and I, Table 2). CD15^+^ human granulocytes and CD56^+^ human NK cells were also more abundant in the bone marrow than in the spleen (Figure 4F and J, Table 2).

Consistent with previous reports, the majority of human cells present in the reconstituted mice were lymphocytes. CD19^+^ B cells accounted for ∼3/4 of the human cells in the spleen and ∼2/3 of the human cells in the bone marrow (Figure 4K and L). Approximately 75% of the CD19^+^ cells in the spleen were IgM^+^IgD^+^ naïve B cells. In the bone marrow, approximately 65% of the CD19^+^ cells were IgM^−^IgD^−^ CD10^+^ pro-B or pre-B cells (Figure 4L). Based on CD34 and CD10 expression, approximately 12% were pro-B cells and 50% were pre-B cells (Table 2). In addition, only ∼4% of human CD19^+^ cells in the bone marrow were naïve B cells.

Compared to B cell reconstitution, the level of T cell reconstitution in the adult NSG mice injected with expanded CD34^+^ CD133^+^ cells was fairly low (Figure 4M–O, Table 2). In the thymus, most of thymocytes were of human origin and were CD4^+^ and CD8^+^, with small proportions of CD4^−^ CD8^−^ or CD4^+^ or CD8^+^ cells, suggesting normal development of human T cells in the thymus. In the spleen, ∼5% of human cells were CD3^+^ T cells. The ratio of CD4 to CD8 T cells was 2 to 1, similar to that in humans.

Taken together, these results show that expanded CD34^+^ CD133^+^ cells are capable of giving rise to different lineages of human blood cells in the adult NSG mice.

## Discussion

We have previously reported expansion of SCID repopulating activity of human cord blood-derived CD133^+^ cells in a medium containing angiopoietin-like protein 5, IGFBP2, SCF, TPO and FGF1 [14]. Here we show that the SCID repopulating activity is correlated with the presence of CD34^+^ CD133^+^ cells in culture. Expansion of CD34^+^ CD133^+^ cells also expands cells capable of mediating long-term multi-lineage hematopoiesis and serial reconstitution in NSG mice. In addition, we found that expanded CD34^+^ CD133^+^ cells give robust reconstitution in the bone marrow, spleen and blood of adult NSG recipients. Here we discuss these observations and highlight their potential applications.

### CD34^+^ CD133^+^ cells measure expansion of hematopoietic stem cells

Many cell surface markers have been identified that define populations enriched for freshly isolated human HSCs, including the CD133^+^ subset of CD34^+^ hematopoietic cells first identified in 1997 by Yin et al. [18]. However much less is known about whether these marker sets retain their predictive value after culture [19]. Total cell number and fold increase in CD34^+^ cells have most commonly been used, and while these correlate with expansion of SRCs the ratio of SRCs/cell changes dramatically during culture. Recently CD34 expression was used in a chemical library screen to identify StemReginin1 as a chemical mediator of HSC expansion [20]. However, in this case as in many others a dramatic (>1100 fold) expansion of CD34 expressing cells led to only a modest ∼16 fold increase in SRC number. Here we report that the use of both CD34 and CD133 led to a much better correlation with SRC number both before and after culture (Figure 1). Our culture method did not lead to nearly such dramatic CD34^+^ cell number increases, perhaps due to the absence of growth factors such as Flt-3 ligand that normally support committed progenitors expressing CD34. We did notice greater variability in CD34^+^ cell expansion, a trend similar to that reported by Delaney et al. who saw CD34^+^ cell number expansion ranging from 36–688 fold with an increase in long term SRC frequency of ∼6 fold [16]. As demonstrated in the discovery of StemReginin 1, surrogate markers for monitoring HSC expansion are extremely valuable for screening compounds that stimulate HSC expansion and could be key to quality control in HSC transplantation.

### Angiopoietin like 5 and IGFBP2 mediate expansion of human HSCs

The stable long-term multi-lineage reconstitution, as well as serial reconstitution with expanded CD34^+^ CD133^+^ cells, which could be assessed in NSG mice but not NOD-scid mice, suggests that these cells contain an expanded subpopulation of long term HSCs. This expanded population also possesses the same SCID repopulating activity as unexpanded CD34^+^ CD133^+^ cells. Limiting dilution analysis showed that similar numbers of expanded and unexpanded CD34^+^ CD133^+^ cells were required to minimally repopulate NSG recipients. These results further support the idea that the angiopoietin-like family of proteins and IGFBP2 are important in promoting expansion of both murine and human HSCs in culture [12], [13], [14]. It is likely that these growth factors mediate HSC survival *in vivo* although to date no direct testing of this hypothesis, for example with inducible knockouts, has been conducted. The recent clinical trial reported by Delaney et al. demonstrated the short-term clinical benefits of using two cord blood samples one of which was expanded to reduce the period of neutropenia following transplantation [21]. However, while the cultured cord blood dominated short-term engraftment, the uncultured cord blood dominated long-term engraftment. This suggests that as yet the optimal culture and transplantation conditions have not been found and other culture systems showing promising results in xenotransplantation studies should be tested in similar clinical trials. The work reported here in combination with the work previously reported by Zhang et al. suggests that the cocktail of SCF, TPO, FGF, IGFBP2 and Angptl5 should be one of those investigated clinically.

### Reconstitution of adult NSG mice with expanded CD34^+^ CD133^+^ cells offers an efficient way to construct humanized mice

Previously, two studies investigated reconstitution of adult NSG mice with purified CD34^+^ cells. In the study by Shultz et al, sub-lethally irradiated adult NSG mice were transferred with mobilized peripheral blood CD34^+^ cells. Hayakawa et al. used busulfan conditioning rather than sub-lethal irradiation and both mobilized peripheral blood CD34^+^ cells and cord blood CD34^+^ cells were used [22], [23]. In these studies ∼50% reconstitution in blood and ∼90% in bone marrow were achieved using 2 million fresh cord blood CD34^+^ cells. This is similar to the levels of reconstitution we achieved with the cultured progeny of 12–25,000 CD34^+^CD133^+^ cells. Where CD34^+^ cells derived from adults were used much lower levels of reconstitution were achieved, possibly reflecting reduced HSC potential with increasing donor age.

Giassi et al. also investigated reconstitution with cultured cord blood CD34^+^ cells [15]. In their study, only <5% reconstitution in the peripheral blood and ∼25% reconstitution in the bone marrow were observed following injection of 1×10^6^ cultured cells derived from ∼27,000 cord blood CD34^+^ cells per recipient. We injected the progeny of a similar small number of cells (12–25,000 CD34^+^CD133^+^ cells) and achieved ∼50% reconstitution in the blood and ∼90% in the bone marrow. This far more robust reconstitution is likely due to the difference in *ex vivo* culture. Our culture method is, to our knowledge, the first method examined to date that generates large numbers robustly reconstituted humanized mice from small numbers of purified HSCs. This technology is valuable to increase cohort sizes of humanized mice without having to use many unrelated donors. The option of using adult NSG mice also eliminates the technical hurdles imposed by reconstitution of neonates making experiments easier to conduct.

We also characterized the development of various human blood lineage cells in NSG recipients following transfer with expanded CD34^+^ CD133^+^ cells. Human myeloid lineage cells, including erythrocytes, platelets, monocytes, granulocytes and dendritic cells, were detected at low levels in recipient mice, similar to those observed in NSG recipients injected with unexpanded HSCs reported by Shultz et al. and Hayakawa et al. [8], [22], [23]. The poor myeloid cell reconstitution is likely due to deficiencies in human cytokines as described in the literature [24], [25] rather than any loss of potential due to *in vitro* culture. In contrast, the lymphoid lineage is robustly reconstituted. Large numbers of B cell precursors are present in the bone marrow and the majority of B cells in the spleen are naïve B cells (CD19^+^IgM^+^IgD^+^). However, many of these cells still express CD10 indicating they may not be fully mature [26]. The latter observation is consistent with the report by Ishikawa et al. showing that many CD19^+^ cells in the spleen expressed IgM but not IgD and that the majority of cells still express CD10 [8]. Compared to B cells, T cell reconstitution is more modest although the ratio of CD4 to CD8 and proportion of different thymocyte populations indicates normal T cell development. The low number of T cells is probably due to the reduced response of human T cells and their precursors to the mouse survival cytokines IL-7 and IL-15 in NSG recipients [24]. Nevertheless, the level of T cell reconstitution in our study was significantly better than that reported by both Hayakawa et al. (0.3%) and Giassi et al., who did not detect any T cells without treating mice with TNF. It seems that HSCs expanded with our culture conditions favor lymphoid cell development in NSG mice, in a manner reminiscent of uncultured cells, whereas HSCs cultured with the method used by Giassi et al. favor the development of myeloid cells possibly due to the presence of Flt3-L, which supports the survival and expansion of myeloid progenitor cells [27].

In summary, we have demonstrated that the SCID repopulating cells we previously reported [14] contain a ∼20× expanded population of HSCs. In addition, we have provided evidence that surface expression of both CD34 and CD133 correlates with the *in vivo* repopulating activity of the expanded HSCs. Finally, we have shown that reconstitution of adult NSG mice with expanded HSCs provides a powerful method to generate large numbers of humanized mice using HSCs from a single cord blood sample. The method reported here represents an important step towards building a generally applicable humanized mouse model for research and offers an improved and robust culture method for expanding human HSCs for clinical follow-up.
